# Realistic Actor-Critic: A framework for balance between value overestimation and underestimation

**DOI:** 10.3389/fnbot.2022.1081242

**Published:** 2023-01-09

**Authors:** Sicen Li, Qinyun Tang, Yiming Pang, Xinmeng Ma, Gang Wang

**Affiliations:** ^1^College of Mechanical and Electrical Engineering, Harbin Engineering University, Harbin, China; ^2^Science and Technology on Underwater Vehicle Laboratory, Harbin Engineering University, Harbin, China; ^3^College of Shipbuilding Engineering, Harbin Engineering University, Harbin, China

**Keywords:** reinforcement learning (RL), robot control, estimation bias, exploration-exploitation dilemma, uncertainty

## Abstract

**Introduction:**

The value approximation bias is known to lead to suboptimal policies or catastrophic overestimation bias accumulation that prevent the agent from making the right decisions between exploration and exploitation. Algorithms have been proposed to mitigate the above contradiction. However, we still lack an understanding of how the value bias impact performance and a method for efficient exploration while keeping stable updates. This study aims to clarify the effect of the value bias and improve the reinforcement learning algorithms to enhance sample efficiency.

**Methods:**

This study designs a simple episodic tabular MDP to research value underestimation and overestimation in actor-critic methods. This study proposes a unified framework called Realistic Actor-Critic (RAC), which employs Universal Value Function Approximators (UVFA) to simultaneously learn policies with different value confidence-bound with the same neural network, each with a different under overestimation trade-off.

**Results:**

This study highlights that agents could over-explore low-value states due to inflexible under-overestimation trade-off in the fixed hyperparameters setting, which is a particular form of the exploration-exploitation dilemma. And RAC performs directed exploration without over-exploration using the upper bounds while still avoiding overestimation using the lower bounds. Through carefully designed experiments, this study empirically verifies that RAC achieves 10x sample efficiency and 25% performance improvement compared to Soft Actor-Critic in the most challenging Humanoid environment. All the source codes are available at https://github.com/ihuhuhu/RAC.

**Discussion:**

This research not only provides valuable insights for research on the exploration-exploitation trade-off by studying the frequency of policies access to low-value states under different value confidence-bounds guidance, but also proposes a new unified framework that can be combined with current actor-critic methods to improve sample efficiency in the continuous control domain.

## 1. Introduction

Reinforcement learning is a major tool to realize intelligent agents that can be autonomously adaptive to the environment (Namiki and Yokosawa, 2021; Yu, 2018; Fukuda, 2020). However, current reinforcement learning techniques still suffer from requiring a huge amount of interaction data, which could result in unbearable costs in real-world applications (Karimpanal and Bouffanais, 2018; Levine et al., 2018; Sutton and Barto, 2018; Dulac-Arnold et al., 2020). This study aims to mitigate this problem by better balancing exploration and exploitation.

Undesirable overestimation bias and accumulation of function approximation errors in temporal difference methods may lead to sub-optimal policy updates and divergent behaviors (Thrun and Schwartz, 1993; Pendrith and Ryan, 1997; Fujimoto et al., 2018; Chen et al., 2022). Most model-free off-policy RL methods learn approximate lower confidence bound of Q-function (Fujimoto et al., 2018; Kuznetsov et al., 2020; Lan et al., 2020; Chen et al., 2021; Lee et al., 2021) to avoid overestimation by introducing underestimation bias. However, if the lower bound has a spurious maximum, it will discourage policy to explore potentially higher uncertain regions, resulting in stochastic local-maximum and causing pessimistic underexploration (Ciosek et al., 2019). Moreover, directionally uninformed (Ciosek et al., 2019) policies, such as Gaussian policies, cannot avoid fully explored wasteful actions.

Optimistic exploration methods (Brafman and Tennenholtz, 2002; Kim et al., 2019; Pathak et al., 2019) learn upper confidence bounds of the Q-function from an epistemic uncertainty estimate. These methods are directionally informed and encourage policy to execute overestimated actions to help agents escape local optimum. However, such upper confidence bound might cause an agent to over-explore low-value regions. In addition, it increases the risk of value overestimation since transitions with high uncertainty may have higher function approximation errors to make the value overestimated. To avoid the above problems, one must carefully adjust hyperparameters and control the bias to keep the value at a balance point between lower and higher bounds: supporting stable learning while providing good exploration behaviors. We highlight that this balance is a particular form of the exploration–exploitation dilemma (Sutton and Barto, 2018). Unfortunately, most prior works have studied the overestimation and pessimistic underexploration in isolation and have ignored the under-/overestimation trade-off aspect.

We formulate the Realistic Actor-Critic (RAC), whose main idea is to learn together values and policies with different trade-offs between underestimation and overestimation in the same network. Policies guided by lower bounds control overestimation bias to provide consistency and stable convergence. Each policy guided by different upper bounds provides a unique exploration strategy to generate overestimated actions, so that the policy family can directionally explore overestimated state-action pairs uniformly and avoid over-exploration. All transitions are stored in a shared replay buffer, and all policies benefit from them to escape spurious maximum. Such a family of policies is jointly parameterized with the Universal Value Function Approximators (UVFA) (Schaul et al., 2015). The learning process can be considered as a set of auxiliary tasks (Badia et al., 2020b; Lyle et al., 2021) that help build shared state representations and sills.

However, learning such policies with diverse behaviors in a single network is challenging since policies vary widely in behavior. We introduce punished Bellman backup, which calculates uncertainty as punishment to correct value estimations. Punished Bellman backup provides fine-granular estimation control to make value approximation shift smoothly between upper and lower bounds, allowing for more efficient training. An ensemble of critics is learned to produce well-calibrated uncertainty estimations (i.e., standard deviation) on unseen samples (Amos et al., 2018; Pathak et al., 2019; Lee et al., 2021). We show empirically that RAC controls the standard deviation and the mean of value estimate bias to close to zero for most of the training. Benefiting from well-bias control, critics are trained with a high update-to-data (UTD) ratio (Chen et al., 2021) to improve sample efficiency significantly.

Empirically, we implement RAC with SAC (Haarnoja et al., 2018) and TD3 (Fujimoto et al., 2018) in continuous control benchmarks (OpenAI Gym Brockman et al., 2016, MuJoCo Todorov et al., 2012). Results demonstrate that RAC significantly improves the performance and sample efficiency of SAC and TD3. RAC outperforms the current state-of-the-art algorithms (MBPO Janner et al., 2019, REDQ Chen et al., 2021, and TQC Kuznetsov et al., 2020), achieving state-of-the-art sample efficiency on the Humanoid benchmark. We perform ablations and isolate the effect of the main components of RAC on performance. Moreover, we perform hyperparameter ablations and demonstrate that RAC is stable in practice. The higher sample efficiency allows RAC to facilitate further applications of the RL algorithm in automatic continuous control.

This study makes the following contributions:

- Highlighting that agents could over-explore low-value states due to inflexible under-/overestimation trade-off in the fixed hyperparameters setting, and it is a particular form of the exploration–exploitation dilemma;
- Defining a unified framework called Realistic Actor-Critic (RAC), which employs Universal Value Function Approximators (UVFA) to simultaneously learn policies with different value confidence-bond with the same neural network, each with a different under-/overestimation trade-off;
- Experimental evidence that the performance and sample efficiency of the proposed method are better than state-of-the-art methods on continuous control tasks.

The study is organized as follows. Section 2 describes related works and their results. Section 3 describes the problem setting and preliminaries of RL. Section 4 explains the under-/overestimation trade-off. Section 5 introduces the punished Bellman backup and RAC algorithm. Section 6 presents experimental results that show the sample efficacy and final performance of RAC. Finally, Section 7 presents our conclusions.

## 2. Related works

### 2.1. Underestimation and overestimation of Q-function

The maximization update rule in Q-learning has been shown to suffer from overestimation bias which is cited as the reason for nonlinear function approximation fails in RL (Thrun and Schwartz, 1993).

Minimizing the value ensemble is a standard method to deal with overestimation bias. Double DQN (Van Hasselt et al., 2016) was shown to be effective in alleviating this problem for discrete action spaces. Clipped double Q-learning (CDQ) (Fujimoto et al., 2018) took the minimum value between a pair of critics to limit overestimation. Maxmin Q-learning (Lan et al., 2020) mitigated the overestimation bias by using a minimization over multiple action-value estimates. However, minimizing a Q-function set cannot filter out abnormally small values, which causes undesired pessimistic underexploration problem (Ciosek et al., 2019). Using minimization to control overestimation is coarse and wasteful as it ignores all estimates except the minimal one (Kuznetsov et al., 2020).

REDQ (Chen et al., 2021) proposed in-target minimization, which used a minimization across a random subset of Q-functions from the ensemble to alleviate the above problems. REDQ (Chen et al., 2021) showed that their method reduces the standard deviation of the Q-function bias to close to zero for most of the training. Truncated Quantile Critics (TQC) (Kuznetsov et al., 2020) truncated the right tail of the distributional value ensemble by dropping several of the topmost atoms to control overestimation. Weighted bellman backup (Lee et al., 2021) and uncertainty-weighted actor-critic (Wu et al., 2021) prevent error propagation (Kumar et al., 2020) in Q-learning by reweighing sample transitions based on uncertainty estimations from the ensembles (Lee et al., 2021) or Monte Carlo dropout (Wu et al., 2021). AdaTQC (Kuznetsov et al., 2021) proposed an auto mechanism for controlling overestimation bias. Unlike prior works, our work does not reweight sample transitions but directly adds uncertainty estimations to punish the target value.

The effect of underestimation bias on learning efficiency is environment-dependent (Lan et al., 2020). Therefore, choosing suitable parameters to balance under- and overestimating for entirely different environments may be hard. This work propose to solve this problem by learning about optimistic and pessimistic policy families.

### 2.2. Ensemble methods

In deep learning, ensemble methods are often used to solve the two key issues, uncertainty estimations (Wen et al., 2020; Abdar et al., 2021) and out-of-distribution robustness (Dusenberry et al., 2020; Havasi et al., 2020; Wenzel et al., 2020). In reinforcement learning, using an ensemble to enhance value function estimation was widely studied, such as averaging a Q-ensemble (Anschel et al., 2017; Peer et al., 2021), bootstrapped actor-critic architecture (Kalweit and Boedecker, 2017; Zheng et al., 2018), calculating uncertainty to reweight sample transitions (Lee et al., 2021), minimization over ensemble estimates (Lan et al., 2020; Chen et al., 2021), and updating the actor with a value ensemble (Kuznetsov et al., 2020; Chen et al., 2021). MEPG (He et al., 2021) introduced a minimalist ensemble consistent with Bellman update by utilizing a modified dropout operator.

A high-level policy can be distilled from a policy ensemble (Chen and Peng, 2019; Badia et al., 2020a) by density-based selection (Saphal et al., 2020), selection through elimination (Saphal et al., 2020), choosing the action that max all Q-functions (Jung et al., 2020; Parker-Holder et al., 2020; Lee et al., 2021), Thompson sampling (Parker-Holder et al., 2020), and sliding-window UCBs (Badia et al., 2020a). Leveraging uncertainty estimations of the ensemble (Osband et al., 2016; Kalweit and Boedecker, 2017; Zheng et al., 2018) simulated training different policies with a multi-head architecture independently to generate diverse exploratory behaviors. Ensemble methods were also used to learn joint state presentation to improve sample efficiency. There were two main methods: multi-heads (Osband et al., 2016; Kalweit and Boedecker, 2017; Zheng et al., 2018; Goyal et al., 2019) and UVFA (Schaul et al., 2015; Badia et al., 2020a,b). This study uses uncertainty estimation to reduce value overestimation bias, a simple max operator to get the best policy, and learning joint state presentation with UVFA.

### 2.3. Optimistic exploration

Pessimistic initialization (Rashid et al., 2020) and a learning policy that maximizes a lower confidence bound value could suffer a pessimistic underexploration problem (Ciosek et al., 2019). Optimistic exploration is a promising solution to ease the above problem by applying the principle of optimism in the face of uncertainty (Brafman and Tennenholtz, 2002). Disagreement (Pathak et al., 2019) and EMI (Kim et al., 2019) considered uncertainty as intrinsic motivation to encourage agents to explore the high-uncertainty areas of the environment. Uncertainty punishment proposed in this study can also be a particular intrinsic motivation. Different from studies of Pathak et al. (2019) and Kim et al. (2019), which usually choose the weight ≥ 0 to encourage exploration, punished Bellman backup use the weight ≤ 0 to control value bias. SUNRISE (Lee et al., 2021) proposed an optimistic exploration that chooses the action that maximizes upper confidence bound (Chen et al., 2017) of Q-functions. OAC (Ciosek et al., 2019) proposed an off-policy exploration policy that is adjusted to a linear fit of upper bounds to the critic with the maximum Kullback–Leibler (KL) divergence constraining between the exploration policies and the target policy. Most importantly, our work provides a unified framework for the under-/overestimation trade-off.

## 3. Problem setting and preliminaries

In this section, we describe the notations and introduce the concept of maximum entropy RL.

### 3.1. Notation

We consider the standard reinforcement learning notation, with states **s**, actions **a**, reward *r*(**s**, **a**), and dynamics *p*(**s′** ∣ **s**, **a**). The discounted return $R_{t}=\overset{\infty }{\underset{k=0}{\sum }}\gamma^{k}r_{k}$ is the total accumulated rewards from timestep *t*, γ ∈ [0, 1] is a discount factor determining the priority of short-term rewards. The objective is to find the optimal policy π_ϕ_(**s** ∣ **a**) with parameters ϕ, which maximizes the expected return *J*(ϕ) = 𝔼_*p*_π__[*R*_*t*_].

### 3.2. Maximum entropy RL

The maximum entropy objective (Ziebart, 2010) encourages the robustness to noise and exploration by maximizing a weighted objective of the reward and the policy entropy:

(1) $$\begin{array}{l} \pi^{*}=\arg\underset{\pi }{\max}\underset{t}{\sum }{𝔼}_{\text{s}\sim p,\text{a}\sim \pi }\left[r\left(\text{s},\text{a}\right)+\alpha H\left(\pi \left(\cdot |\text{s}\right)\right)\right], \end{array}$$

where α is the temperature parameter used to determine the relative importance of entropy and reward. Soft Actor-Critic (SAC) (Haarnoja et al., 2018) seeks to optimize the maximum entropy objective by alternating between a soft policy evaluation and a soft policy improvement. A parameterized soft Q-function *Q*_θ_(**s**, **a**), known as the critic in actor-critic methods, is trained by minimizing the soft Bellman backup:

(2) $$\begin{array}{l} L_{\text{critic}}\left(\theta \right)={𝔼}_{\tau \sim B}\left[{\left(Q_{\theta }\left(\text{s},\text{a}\right)-y\right)}^{2}\right],y\\ =r-\gamma {𝔼}_{{\text{a}}^{\prime }\sim \pi_{\phi }}\left[Q_{\bar{\theta }}\left({\text{s}}^{\prime },{\text{a}}^{\prime }\right)-\alpha \log\pi_{\phi }\left({\text{a}}^{\prime }|{\text{s}}^{\prime }\right)\right], \end{array}$$

where τ = (**s**, **a**, *r*, **s′**) is a transition, B is a replay buffer, $\bar{\theta }$ are the delayed parameters which are updated by exponential moving average $\bar{\theta }\leftarrow \rho \theta +\left(1-\rho \right)\bar{\theta }$, ρ is the target smoothing coefficient, and *y* is the target value. The target value $Q_{\bar{\theta }}\left({\text{s}}^{\prime },{\text{a}}^{\prime }\right)$ is obtained by using two networks $Q_{\bar{\theta }}^{1}\left({\text{s}}^{\prime },{\text{a}}^{\prime }\right)$ and $Q_{\bar{\theta }}^{2}\left({\text{s}}^{\prime },{\text{a}}^{\prime }\right)$ with minimum operator:

(3) $$\begin{array}{l} Q_{\bar{\theta }}\left({\text{s}}^{\prime },{\text{a}}^{\prime }\right)=min\left(Q_{\bar{\theta }}^{1}\left({\text{s}}^{\prime },{\text{a}}^{\prime }\right),Q_{\bar{\theta }}^{2}\left({\text{s}}^{\prime },{\text{a}}^{\prime }\right)\right). \end{array}$$

The parameterized policy π_ϕ_, known as the actor, is updated by minimizing the following object:

(4) $$\begin{array}{l} L_{\text{actor}}\left(\phi \right)={𝔼}_{\text{s}\sim B,\text{a}\sim \pi_{\phi }}\left[\alpha \log\left(\pi_{\phi }\left(\text{a}|\text{s}\right)\right)-Q_{\theta }\left(\text{a},\text{s}\right)\right]. \end{array}$$

SAC uses an automated entropy adjusting mechanism to update α with the following objective:

(5) $$\begin{array}{l} L_{\text{temp}}\left(\alpha \right)={𝔼}_{\text{s}\sim B,\text{a}\sim \pi_{\phi }}\left[-\alpha \log\pi_{\phi }\left(\text{a}|\text{s}\right)-\alpha \bar{H}\right], \end{array}$$

where $\bar{H}$ is the target entropy.

## 4. Understanding under-/overestimation trade-off

This section briefly discusses the estimation bias issue and empirically shows that a better under-/overestimation trade-off may improve learning performance.

### 4.1. Under-/overestimation trade-off

**Figure 1:**
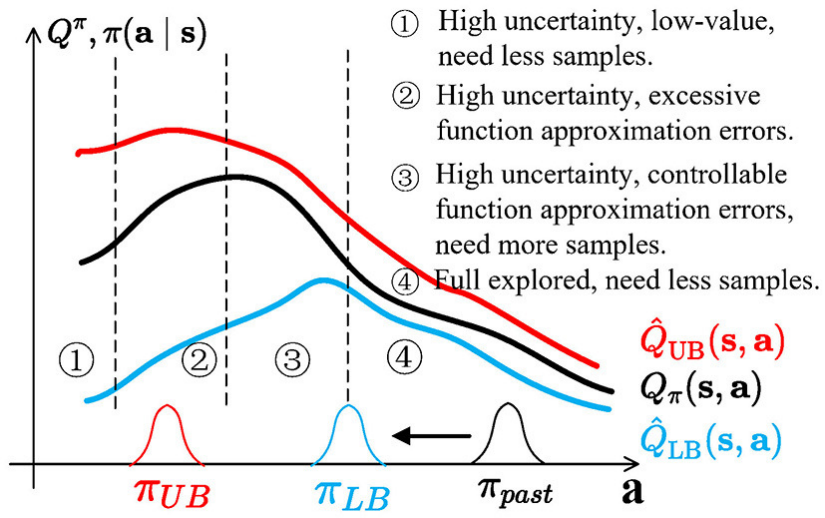
Balance between value underestimation and overestimation in actor-critic methods. The state *s* is fixed. The graph shows *Q*_π_ **(in black)**, which is unknown to the algorithm, estimated lower bound ${\hat{Q}}_{LB}$ **(in blue)**, higher bound ${\hat{Q}}_{UB}$ **(in red)**, two policies, π_*LB*_ **(in blue)** and π_*past*_ **(in black)**, at different time steps of the algorithm, and exploration policies π_*UB*_ **(in red)** for optimistic exploration.

Under-/overestimation trade-off is a special form of the exploration–exploitation dilemma. This is illustrated in Figure 1. At first, the agent starts with a policy π_*past*_, trained with lower bound ${\hat{Q}}_{LB}\left(\text{s},\text{a}\right)$, becoming π_*LB*_. We divide the current action space into four regions:

- High uncertainty, low-value. Highly stochastic regions also have low values; overestimation bias might cause an agent to over-explore a low-value area;
- High uncertainty, excessive errors. This region has high uncertainty but is full of unseen transitions that can have excessive-high approximation errors, which may cause catastrophic overestimation and need fewer samples;
- High uncertainty, controllable errors. This region has high uncertainty and is closer to the π_*LB*_, with controllable approximation errors, and needs more samples;
- Full explored. Since π_*past*_ is gradually updated to π_*LB*_, the area is fully explored and needs less samples.

To prevent catastrophic overestimation bias accumulation, SAC (Haarnoja et al., 2018), TD3 (Fujimoto et al., 2018), and REDQ (Chen et al., 2021) introduce underestimation bias to learn lower confidence bounds of Q-functions, similar to Equation 3. However, directionally uninformed policies, such as gaussian policies, will sample actions located in region 4 with half probability. If the lower bound has a spurious maximum and policies are directionally uninformed (Ciosek et al., 2019), lower bound policy π_*LB*_ may be stuck at the junction of regions 3 and 4. This is wasteful and inefficient, causing pessimistic underexploration.

π_*UB*_, which is used in optimistic exploration methods (Brafman and Tennenholtz, 2002; Kim et al., 2019; Pathak et al., 2019), can encourage agents to execute overestimated actions and explore potential high-value regions with high uncertainty. However, regions with high and overestimated actions, such as region 2, may have excessive function approximation errors. Alternatively, if highly uncertain regions also have low values (like region 1), overestimation bias might cause an agent to over-explore a low-value region.

Ideally, the exploration policies are located in region 3 to provide better exploration behaviors and keep stable updates. There are two ways to achieve this: (1) enforcing a KL constraint between π_*UB*_ and π_*LB*_ (like OAC Ciosek et al., 2019); and (2) balancing $\hat{Q}$ between ${\hat{Q}}_{LB}$ and ${\hat{Q}}_{UB}$, and we call it an under-/overestimation trade-offs.

However, in practical applications, *Q*_π_ is unknown, and it is not easy to tune to ideal conditions through constant hyperparameters.

### 4.2. A simple MDP

**Figure 2:**
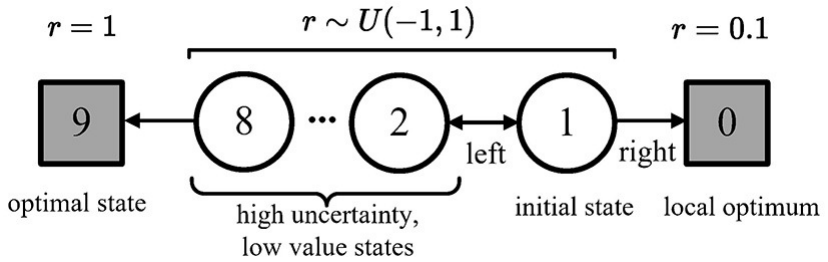
A simple episodic MDP (Lan et al., 2020), adapted from Figure 6.5 in the study of Sutton and Barto (2018). This MDP has two terminal states: state 9 and state 0. Every episode starts from state 1, which has two actions: **Left** and **Right**. The MDP is deterministic. Once the agent takes into any states, the MDP will reward back: *r* = 0.1 for terminal states 0, *r* = 1 for terminal states 9, and a reward *r* ~ *U*(−1, 1) for non-terminal states 1–8. State 9 is the optimal state, state 0 is a local optimum, and states 1–8 are the high-uncertainty and low-value states.

We show this effect in a simple Markov decision process (MDP), as shown in Figure 2. Any state's optimal policy is the left action. If the agent wants to go to state 9, it must go through states 1–8 with high uncertainty and low values.

In the experiment, we used a discount factor γ = 0.9; a replay buffer with size 5, 000; a Boltzmann policy with *temperature* = 0.1; tabular action values with uniform noisy respect to a Uniform distribution *U*(−0.1, 0.1), initialized with a Uniform distribution *U*(−5, 5); and a learning rate of 0.01 for all algorithms.

**Figure 3:**
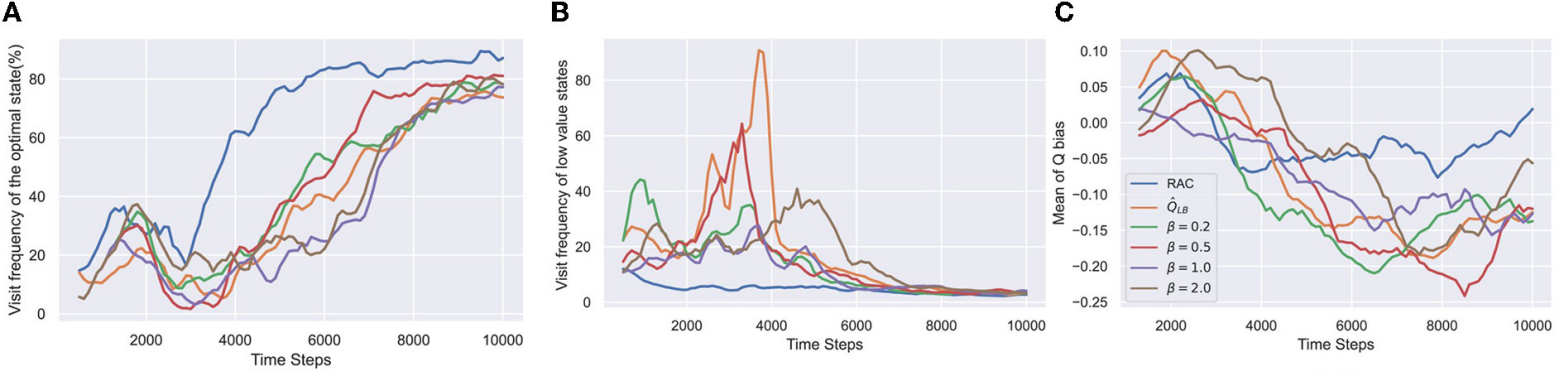
Results of the simple MDP. In-target minimization target from REDQ is used as ${\hat{Q}}_{LB}$. ${\hat{Q}}_{b}=mean\left({\hat{Q}}_{LB}^{\prime }\right)+\beta standarddeviation\left({\hat{Q}}_{LB}^{\prime }\right)$ can perform optimistic exploration. β is a key parameter to control value bias. If β = 0, ${\hat{Q}}_{b}$ is equal to ${\hat{Q}}_{LB}$. As β increases, ${\hat{Q}}_{b}$ gradually approaches ${\hat{Q}}_{UB}$. The horizontal axis indicates the number of time steps. **(A)** Visit frequency of the optimal state is the ratio of the frequency of visiting the optimal state among all termination states. The higher the value, the lower the probability that the agent is stuck in a local optimum. **(B)** Visit frequency of low-value states is the ratio of the visit frequency of low-value state 2–8 and the optimal state 9. The lower the value, the fewer steps the agent wastes in low-value states. This value has been subtracted by 7, as the minimum step size to reach the optimum state is seven. **(C)** Q bias measures the difference between the estimated Q values and true Q values. All results are estimated by the Monte Carlo method and averaged over eight seeds.

The results in Figure 3 verify our hypotheses in Section 4.1. All algorithms converge, but each has a different convergence speed. ${\hat{Q}}_{LB}$ underestimates too much and converges to a suboptimal policy in the early learning stage, causing slow convergence. For β = 0.5 and 1.0, optimistic exploration drives the agent to escape the local optimum and learn faster. However, $\hat{Q}$ overestimates too much for β = 2.0, significantly impairing the convergence speed of the policy. In addition, no matter what parameter β takes, the agent still over-explores low-value states at different time steps (see Figure 3).

RAC avoids over-exploration in low-value states and is the fastest to converge to the optimal policy. Furthermore, RAC maintains the Q bias close to zero without catastrophic overestimation throughout the learning process, indicating that RAC keeps an outstanding balance between underestimation and overestimation.

## 5. Realistic Actor-Critic

We present Realistic Actor-Critic (RAC), which can be used in conjunction with the most modern off-policy actor-critic RL algorithms in principle, such as SAC (Haarnoja et al., 2018) and TD3 (Fujimoto et al., 2018). We describe only the SAC version of RAC (RAC-SAC) in the main body for the exposition. The TD3 version of RAC (RAC-TD3) follows the same principles and is fully described in Appendix B.

### 5.1. Punished Bellman backup

Punished Bellman backup is a variant of soft Bellman backup (Equation 2). The idea is to maintain an ensemble of *N* soft Q-functions *Q*_θ_*i*__(**s**, **a**), where θ_*i*_ denotes the parameters of the *i* − *th* soft Q-function, which are initialized randomly and independently for inducing an initial diversity in the models (Osband et al., 2016), but updated with the same target.

Given a transition τ_*t*_, punished Bellman backup considers following punished target *y*:

(6) $$\begin{array}{l} \begin{array}{l} y=r_{t}+\gamma {𝔼}_{{\text{a}}^{\prime }\sim \pi_{\phi }}[{\bar{Q}}_{\bar{\theta }}\left({\text{s}}^{\prime },{\text{a}}^{\prime }\right)-\beta ŝ\left(Q_{\bar{\theta }}\left({\text{s}}^{\prime },{\text{a}}^{\prime }\right)\right)\\ -\alpha \log\pi_{\phi }\left({\text{a}}^{\prime }|{\text{s}}^{\prime }\right)], \end{array} \end{array}$$

where ${\bar{Q}}_{\bar{\theta }}\left(\text{s},\text{a}\right)$ is the sample mean of the target Q-functions and $ŝ\left(Q_{\bar{\theta }}\left(\text{s},\text{a}\right)\right)$ is the sample standard deviation of target Q-functions with bessel's correction (Warwick and Lininger, 1975). Punished Bellman backup uses $ŝ\left(Q_{\bar{\theta }}\left(\text{s},\text{a}\right)\right)$ as uncertainty estimation to punish value estimation. β ≥ 0 is the weighting of the punishment. Note that we do not propagate gradient through the uncertainty $ŝ\left(Q_{\bar{\theta }}\left(\text{s},\text{a}\right)\right)$.

We write $Q_{\text{s}\text{a}}^{i}$ instead of *Q*_θ_*i*__(**s**, **a**) and $Q_{{\text{s}}^{\prime }{\text{a}}^{\prime }}^{i}$ instead of $Q_{\theta_{i}}\left({\text{s}}^{\prime },{\text{a}}^{\prime }\right)$ for compactness. Assuming each Q-function has random approximation error $e_{\text{s}\text{a}}^{i}$ (Thrun and Schwartz, 1993; Lan et al., 2020; Chen et al., 2021), which is a random variable belonging to some distribution,

(7) $$\begin{array}{l} Q_{\text{s}\text{a}}^{i}=Q_{\text{s}\text{a}}^{*}+e_{\text{s}\text{a}}^{i}, \end{array}$$

where $Q_{\text{s}\text{a}}^{*}$ is the ground truth of Q-functions. *M* is the number of actions applicable at state **s′**. The estimation bias *Z*_*MN*_ for a transition τ_*t*_ is defined as

(8) $$\begin{array}{l} \begin{array}{l} Z_{MN}\overset{\text{def}}{=}\left[r+\gamma \underset{{\text{a}}^{\prime }}{\max}\left(Q_{{\text{s}}^{\prime }{\text{a}}^{\prime }}^{mean}-\beta Q_{{\text{s}}^{\prime }{\text{a}}^{\prime }}^{std}\right)\right]-\left(r+\gamma \underset{{\text{a}}^{\prime }}{\max}Q_{{\text{s}}^{\prime }{\text{a}}^{\prime }}^{*}\right)\\ \;=\gamma \left[\underset{{\text{a}}^{\prime }}{\max}\left(Q_{{\text{s}}^{\prime }{\text{a}}^{\prime }}^{mean}-\beta Q_{{\text{s}}^{\prime }{\text{a}}^{\prime }}^{std}\right)-\underset{{\text{a}}^{\prime }}{\max}Q_{{\text{s}}^{\prime }{\text{a}}^{\prime }}^{*}\right], \end{array} \end{array}$$

where

(9) $$\begin{array}{l} \begin{array}{l} Q_{{\text{s}}^{\prime }{\text{a}}^{\prime }}^{mean}\approx \frac{1}{N}\overset{N}{\underset{i=1}{\sum }}Q_{{\text{s}}^{\prime }{\text{a}}^{\prime }}^{i}=\frac{1}{N}\overset{N}{\underset{i=1}{\sum }}\left(Q_{{\text{s}}^{\prime }{\text{a}}^{\prime }}^{*}+e_{{\text{s}}^{\prime }{\text{a}}^{\prime }}^{i}\right)=Q_{{\text{s}}^{\prime }{\text{a}}^{\prime }}^{*}\\ \;+\frac{1}{N}\overset{N}{\underset{i=1}{\sum }}e_{{\text{s}}^{\prime }{\text{a}}^{\prime }}^{i}=Q_{{\text{s}}^{\prime }{\text{a}}^{\prime }}^{*}+{ē}_{{\text{s}}^{\prime }{\text{a}}^{\prime }}, \end{array} \end{array}$$

(10) $$\begin{array}{l} \begin{array}{l} Q_{{\text{s}}^{\prime }{\text{a}}^{\prime }}^{std}\approx \sqrt{\frac{1}{N-1}\overset{N}{\underset{i=1}{\sum }}{\left(Q_{{\text{s}}^{\prime }{\text{a}}^{\prime }}^{i}-Q_{{\text{s}}^{\prime }{\text{a}}^{\prime }}^{mean}\right)}^{2}}\\ \;=\sqrt{\frac{1}{N-1}\overset{N}{\underset{i=1}{\sum }}{\left(Q_{{\text{s}}^{\prime }{\text{a}}^{\prime }}^{*}+e_{{\text{s}}^{\prime }{\text{a}}^{\prime }}^{i}-Q_{{\text{s}}^{\prime }{\text{a}}^{\prime }}^{*}+{ē}_{{\text{s}}^{\prime }{\text{a}}^{\prime }}\right)}^{2}}\\ \;=\sqrt{\frac{1}{N-1}\overset{N}{\underset{i=1}{\sum }}{\left(e_{{\text{s}}^{\prime }{\text{a}}^{\prime }}^{i}-{ē}_{{\text{s}}^{\prime }{\text{a}}^{\prime }}\right)}^{2}}=ŝ\left(e_{{\text{s}}^{\prime }{\text{a}}^{\prime }}\right). \end{array} \end{array}$$

Then,

(11) $$\begin{array}{l} Z_{MN}\approx \gamma \left[\underset{{\text{a}}^{\prime }}{\max}\left(Q_{{\text{s}}^{\prime }{\text{a}}^{\prime }}^{*}+{ē}_{{\text{s}}^{\prime }{\text{a}}^{\prime }}-\beta ŝ\left(e_{{\text{s}}^{\prime }{\text{a}}^{\prime }}\right)\right)-\underset{{\text{a}}^{\prime }}{\max}Q_{{\text{s}}^{\prime }{\text{a}}^{\prime }}^{*}\right]. \end{array}$$

If one could choose $\beta =\frac{{ē}_{{\text{s}}^{\prime }{\text{a}}^{\prime }}}{ŝ\left(e_{{\text{s}}^{\prime }{\text{a}}^{\prime }}\right)}$, $Q_{\text{s}\text{a}}^{i}$ will be resumed to $Q_{\text{s}\text{a}}^{*}$, then *Z*_*MN*_ can be reduced to near 0. However, it's hard to adjust a suitable constant β for various state-action pairs actually. We develop vanilla RAC, which uses a constant β Appendix B.3, to research this problem.

For β = 0, the update is simple average Q-learning which causes overestimation bias (Chen et al., 2021). As β increases, increasing penalties $Q_{{\text{s}}^{\prime }{\text{a}}^{\prime }}^{std}$ decrease *E*[*Z*_*MN*_] gradually and encourage Q-functions to transit smoothly from higher bounds to lower bounds.

### 5.2. Realistic Actor-Critic agent

We demonstrate how to use punished Bellman backup to incorporate various bounds of value approximations into a full agent that maintains diverse policies, each with a different under-/overestimation trade-off. The pseudocode for RAC-SAC is shown in Algorithm 1.

**Algorithm 1:** RAC: SAC version.

1:	Initialize actor network ϕ, *N* critic networks θ_*i*_, *i* = 1, …, *N*, temperature network ψ, empty replay buffer B, target network $\bar{\theta_{i}}\leftarrow \theta_{i}$, for *i* = 1, 2, …, *N*, uniform distribution *U*_1_ and *U*_2_
2:	**for** each iteration **do**
3:	// optimistic exploration
4:	execute an action *a* ~ π_ϕ_(·∣*s*, β), β~*U*_2_.
5:	Observe reward *r*_*t*_, new state *s*′
6:	Store transition tuple $B\leftarrow B\cup \left\{\left(s,a,r_{t},s^{\prime }\right)\right\}$
7:	**for** *G* updates **do**
8:	// update critics via punished bellman backup
9:	Sample random minibatch:
10:	${\left\{\tau_{j}\right\}}_{j=1}^{B}\sim B$, ${\left\{\beta_{m}\right\}}_{m=1}^{B}\sim U_{1}$
11:	Compute the Q target (Equation 13)
12:	**for** *i* = 1, …, *N* **do**
13:	Update θ_*i*_ by minimize $L_{\text{critic}}^{\text{RAC}}$ (Equation 13)
14:	Update target networks:
15:	$\bar{\theta_{i}}\leftarrow \rho \bar{\theta_{i}}+\left(1-\rho \right)\theta_{i}$
16:	// update actors and temperatures according to *U*_1_
17:	Update ϕ by minimize $L_{\text{actor}}^{\text{RAC-SAC}}$ (Equation 14)
18:	Update ψ by minimize $L_{\text{temp}}^{\text{RAC}}$ (Equation 12)
-

RAC uses UVFA (Schaul et al., 2015) to extend the critic and actor as *Q*_θ_*i*__(**s**, **a**, β) and $\pi_{\phi }\left(\cdot |{\text{s}}^{\prime },\beta \right)$, *U*_1_ is a uniform training distribution U[0,a], *a* is a positive real number, and β ~ *U*_1_ that generates various bounds of value approximations.

An independent temperature network α_ψ_ parameterized by ψ is used to accurately adjust the temperature with respect to β, which can improve the performance of RAC. Then, the objective (Equation 5) becomes:

(12) $$\begin{array}{l} \begin{array}{l} L_{\text{temp}}^{\text{RAC}}\left(\psi \right)={𝔼}_{\text{s}\sim B,\text{a}\sim \pi_{\phi },\beta \sim U_{1}}[-\alpha_{\psi }\left(\beta \right)\log\pi_{\phi }\left(\text{a}|\text{s},\beta \right)\\ \;-\alpha_{\psi }\left(\beta \right)\bar{H}]. \end{array} \end{array}$$

The extended Q-ensemble use punished Bellman backup to simultaneously approximate a soft Q-function family:

(13) $$\begin{array}{l} L_{\text{critic}}^{\text{RAC}}\left(\theta_{i}\right)={𝔼}_{\tau \sim B,\beta \sim U_{1}}\left[{\left(Q_{\theta_{i}}\left(\text{s},\text{a},\beta \right)-y\right)}^{2}\right],\\ y=r+\gamma {𝔼}_{{\text{a}}^{\prime }\sim \pi_{\phi }}[{\bar{Q}}_{\bar{\theta }}\left({\text{s}}^{\prime },{\text{a}}^{\prime },\beta \right)-\beta ŝ\left(Q_{\bar{\theta }}\left({\text{s}}^{\prime },{\text{a}}^{\prime },\beta \right)\right)\\ -\alpha_{\psi }\left(\beta \right)\log\pi_{\phi }\left({\text{a}}^{\prime }|{\text{s}}^{\prime },\beta \right)] \end{array}$$

where ${\bar{Q}}_{\bar{\theta }}\left(\text{s},\text{a},\beta \right)$ is the sample mean of target Q-functions and $ŝ\left(Q_{\bar{\theta }}\left(\text{s},\text{a},\beta \right)\right)$ is the corrected sample standard deviation of target Q-functions.

The extended policy π_ϕ_ is updated by minimizing the following object:

(14) $$\begin{array}{l} L_{\text{actor}}^{\text{RAC-SAC}}\left(\phi \right)={𝔼}_{\text{s}\sim B,\beta \sim U_{1}}[{𝔼}_{\text{a}\sim \pi_{\phi }}[\alpha_{\psi }\left(\beta \right)\log\left(\pi_{\phi }\left(\text{a}|\text{s},\beta \right)\right)\\ \;-{\bar{Q}}_{\theta }\left(\text{a},\text{s},\beta \right)]], \end{array}$$

where ${\bar{Q}}_{\theta }\left(\text{a},\text{s},\beta \right)$ is the sample mean of Q-functions.

A larger UTD ratio *G* improves sample utilization. We find that a smaller replay buffer capacity slightly improves the sample efficiency of RAC in Section 6.5.

Note that we find that applying different samples, which are generated by binary masks from the Bernoulli distribution (Osband et al., 2016; Lee et al., 2021), to train each Q-function will not improve RAC performance in our experiments; therefore, RAC does not apply this method.

#### 5.2.1. RAC circumvents direct adjustment

RAC leaners with a distribution of β instead of a constant β. One could evaluate the policy family to find the best β. We employ a discrete number *H* of values ${\left\{\beta_{i}\right\}}_{i=1}^{H}$ (see details in Appendix A.1) to implement a distributed evaluation for computational efficiency and apply the max operator to get best β.

#### 5.2.2. Optimistic exploration

When interacting with the environment, we propose to sample β uniformly from a uniform explore distribution $U_{2}=U\left[0,b\right]$, where *b* < *a* is a positive real number, to get optimistic exploratory behaviors to avoid pessimistic underexploration (Ciosek et al., 2019).

### 5.3. How RAC solves the under-/overestimation trade-off

Similar to the idea of NGU (Badia et al., 2020b), RAC decouples exploration and exploitation policies. RAC uses UVFA to simultaneously learn policies with the same neural network, each with different trade-offs between underestimation and overestimation. Using UVFA to learn different degrees of confidence bounds allows us to learn a powerful representation and set of skills that can be quickly transferred to the expected policy. With punished Bellman backup, RAC has a larger number of policies and values that change smoothly, allowing for more efficient training.

**Figure 4:**
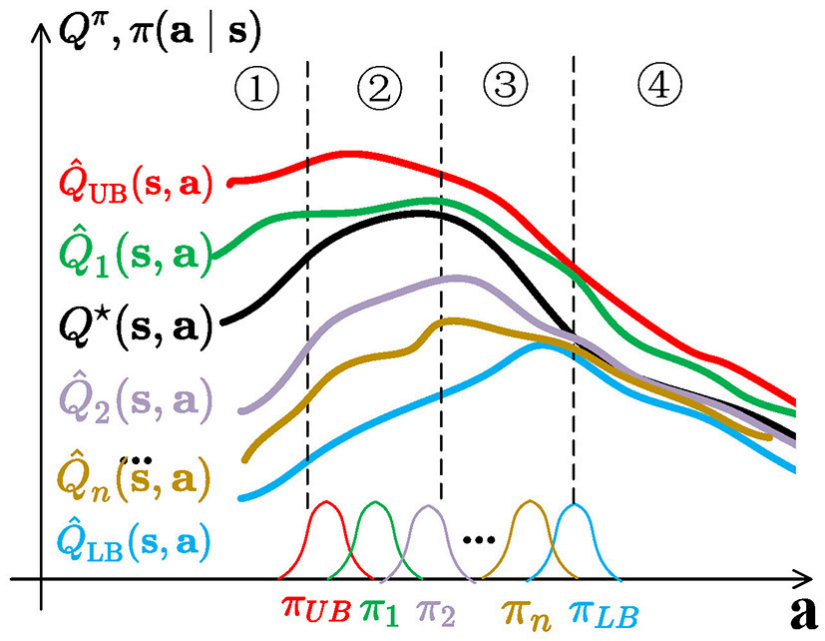
Visualization of RAC. The serial numbers in the figure correspond to Section 4.1 and Figure 1. For better illustration, $\hat{Q}$ is discretized. In fact, ${\hat{Q}}_{n}$ learned by RAC is infinite and changes continuously. *Q*^⋆^(**s**, **a**) is the optimal Q-function that is unknown. Q-functions are distributed between ${\hat{Q}}_{UB}$ and ${\hat{Q}}_{LB}$ and their policies are distributed between π_*UB*_ and π_*LB*_. π_*UB*_, π_1_, and π_2_ are used as exploration policies.

This is illustrated in Figure 4. Q-functions that are close to ${\hat{Q}}_{LB}$ (like ${\hat{Q}}_{n}$) control overestimation bias to provide consistency and stable convergence. Exploration policies (such as π_*UB*_, π_1_, and π_2_) are far from the spurious maximum of ${\hat{Q}}_{LB}$ and ${\hat{Q}}_{n}$, and overestimated actions sampled from them located in regions 1, 2, and 3 lead to a quick correction to the critic estimate. All transitions are stored in a shared replay buffer, and all policies benefit from them to escape spurious maximums. Since exploration policies are not symmetric to the mean of π_*LB*_ and π_*n*_, RAC also avoids directional uninformedness.

Although RAC cannot always keep the exploration policies located in region 3, the policy family avoids all behaviors concentrated in region 1 or 2. Exploration behaviors uniformly distribute in regions 1, 2, and 3, preventing over-exploration in any area.

Moreover, such policies could be quite different from a behavior standpoint and generate varied action sequences to visit unseen state-action pairs following the principle of optimism in the face of uncertainty (Chen et al., 2017; Ciosek et al., 2019; Lee et al., 2021).

## 6. Experiments

We designed our experiments to answer the following questions:

- Can the Realistic Actor-Critic outperform state-of-the-art algorithms in continuous control tasks?
- Can the Realistic Actor-Critic better balance between value overestimation and underestimation?
- What is the contribution of each technique in the Realistic Actor-Critic?

### 6.1. Setups

We implement RAC with SAC and TD3 as RAC-SAC and RAC-TD3 (see Appendix B).

The baseline algorithms are REDQ (Chen et al., 2021), MBPO (Janner et al., 2019), SAC (Haarnoja et al., 2018), TD3 (Fujimoto et al., 2018), and TQC (Kuznetsov et al., 2020). All hyperparameters we used for evaluation are the same as those in the original articles. For MBPO (https://github.com/JannerM/mbpo), REDQ (https://github.com/watchernyu/REDQ), TD3 (https://github.com/sfujim/TD3), and TQC (https://github.com/SamsungLabs/tqc_pytorch), we use the authors' code. For SAC, we implement it following the study of Haarnoja et al. (2018), and the results we obtained are similar to previously reported results. TQC20 is a variant of TQC with UTD *G* = 20 for a fair comparison.

We compare baselines on six challenging continuous control tasks (Walker2d, HalfCheetah, Hopper, Swimmer, Ant, and Humanoid) from MuJoCo environments (Todorov et al., 2012) in the OpenAI gym benchmark (Brockman et al., 2016).

The time steps for training instances on Walker2d, Hopper, and Ant are 3 × 10^5^, and 1 × 10^6^ for Humanoid and HalfCheetah. All algorithms explore with a stochastic policy but use a deterministic policy for evaluation similar to those in SAC. We report the mean and standard deviation across eight seeds.

For all algorithms, we use a fully connected network with two hidden layers and 256 units per layer, with Rectified Linear Unit in each layer (Glorot et al., 2011), for both actor and critic. All the parameters are updated by the Adam optimizer (Kingma and Ba, 2014) with a fixed learning rate. All algorithms adopt almost the same NN architecture and hyperparameter.

For all experiments, our learning curves show the total undiscounted return.

Using the Monte Carlo method, we estimate the mean and standard deviation of normalized Q-function bias (Chen et al., 2021) as the main analysis indicators to analyze the value approximation quality (described in Appendix A). The average bias lets us know whether *Q*_θ_ is overestimated or underestimated, while standard deviation measures whether *Q*_θ_ is overfitting.

Sample efficiency (SE) (Chen et al., 2021; Dorner, 2021) is measured by the ratio of the number of samples collected when RAC and some algorithms reach the specified performance. Hopper is not in the comparison object as the performance of algorithms is almost indistinguishable.

### 6.2. Comparative evaluation

#### 6.2.1. OpenAI gym

**Figure 5:**
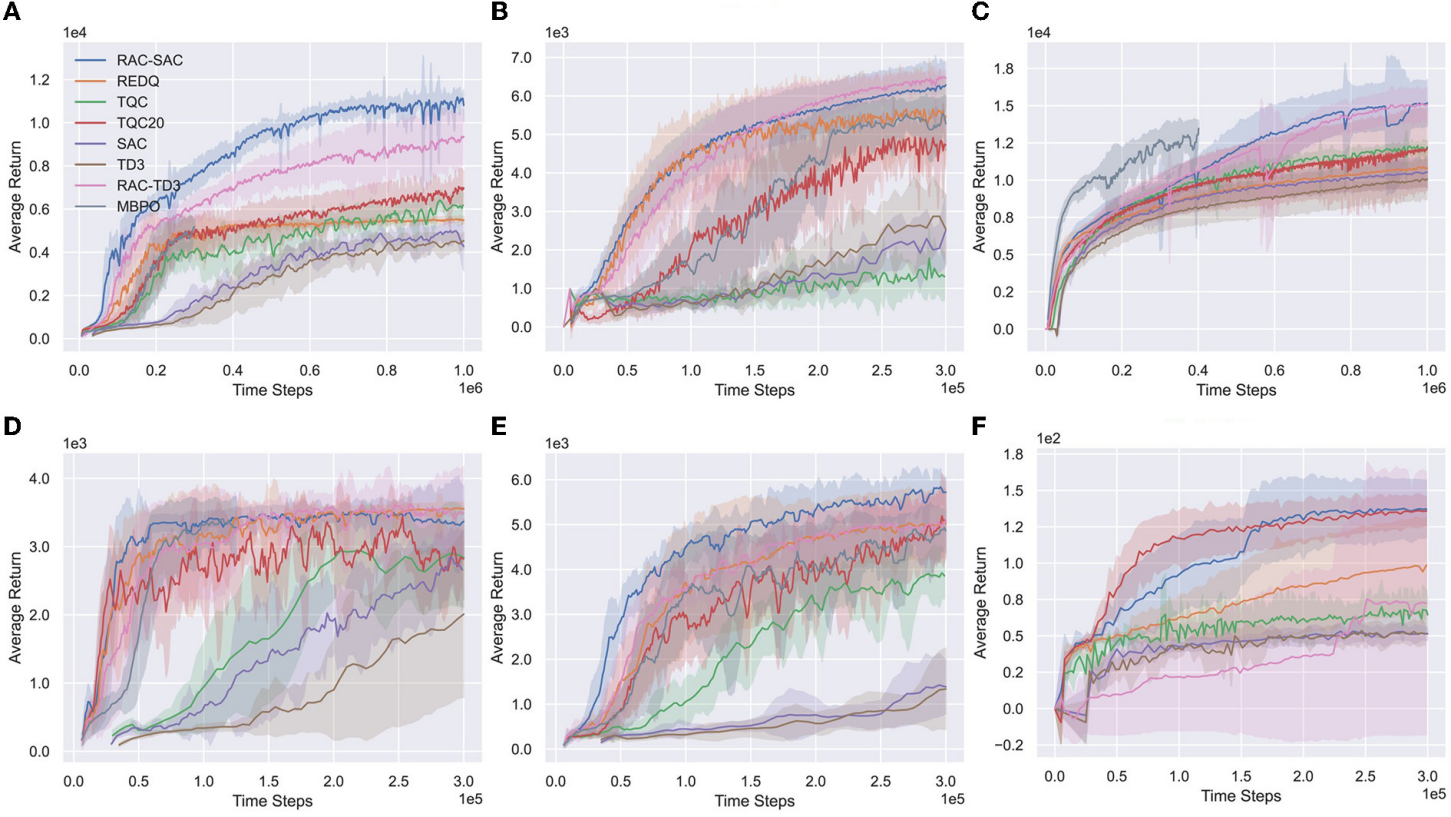
Learning curves on six Mujoco environments. The horizontal axis indicates the number of time steps. The vertical axis shows the average undiscounted return. The shaded areas denote one standard deviation over eight runs. **(A)** Humanold, **(B)** Ant, **(C)** HalfCheetah, **(D)** Hopper, **(E)** Walker2d, and **(F)** Swimmer.

Figure 5 and Table 1 show learning curves and performance comparison. RAC consistently improves the performance of SAC and TD3 across all environments and performs better than other algorithms. In particular, RAC learns significantly faster for Humanoid and has better asymptotic performance for Ant, Walker2d, and HalfCheetah. RAC yields a much smaller variance than SAC and TQC, indicating that the optimistic exploration helps the agents escape from bad local optima.

**Table 1:** Performance on OpenAI gym.

	**RAC-SAC**	**RAC-TD3**	**REDQ**	**MBPO**	**TQC20**	**TD3**	**SAC**	**TQC**
Humanoid	**11,107** ± **475**	9,321 ± 1,126	5,504 ± 120	5,162 ± 350	7,053 ± 857	7,014 ± 643	7,681 ± 1,118	10,731 ± 1,296
Ant	6,283 ± 549	6,470 ± 165	5,475 ± 890	5,281 ± 699	4,722 ± 567	**6,796** ± **277**	6,433 ± 332	6,402 ± 1,371
Walker	**5,860** ± **440**	5,114 ± 489	5,034 ± 711	4,864 ± 488	5,109 ± 696	4,419 ± 1,682	5,249 ± 554	5,821 ± 457
Hopper	3,421 ± 483	3,495 ± 672	**3,563** ± **94**	3,280 ± 455	3,208 ± 538	3,433 ± 321	2,815 ± 585	3,011 ± 866
HalfCheetah	15,717 ± 1,063	15,083 ± 1,113	10,802 ± 1,179	13,477 ± 443	12,123 ± 2,600	14,462 ± 1,982	16,330 ± 323	**17,245** ± **293**
Swimmer	**143** ± **6.8**	71 ± 83	98 ± 31	-	143 ± 9.6	53 ± 8.8	51 ± 4.2	65 ± 5.8

The maximum value for each task is bolded. ± corresponds to a single standard deviation over eight runs. The best results are indicated in bold. Results of SAC, TD3, and TQC are obtained at 6 × 10^6^ time steps for Humanoid and HalfCheetah and 3 × 10^6^ time steps for other environments. Results of RAC, REDQ, and TQC20 are obtained at 1 × 10^6^ time steps for Humanoid and HalfCheetah and 3 × 10^5^ time steps for other environments. Results of MBPO are obtained at 3 × 10^5^ time steps for Ant, Humanoid, and Walker2d, 4 × 10^5^ for HalfCheetah and 1.25 × 10^5^ for Hopper.

#### 6.2.2. Sample-efficiency comparison

Table 2 shows the sample-efficiency comparison with baselines. Compared with TQC, RAC-SAC reaches 3,000 and 6,000 for Ant with 16.79x and 12.31x sample efficiency, respectively. RAC-SAC performs 1.5x better than REDQ halfway through training and 1.8x better at the end of training in Walker and Humanoid. They show that a better under-/overestimation trade-off can achieve better sample-efficiency performance than the MuJoCo environments' state-of-the-art algorithms.

**Table 2:** Sample-efficiency comparison.

	**RAC-SAC**	**REDQ**	**MBPO**	**TQC**	**TQC20**	**REDQ/RAC-SAC**	**MBPO/RAC-SAC**	**TQC/RAC-SAC**	**TQC20/RAC-SAC**
Humanoid at 2,000	63 K	109 K	154 K	145 K	147 K	1.73	2.44	2.30	2.33
Humanoid at 5,000	134 K	250 K	295 K	445 K	258 K	1.87	2.20	3.32	1.93
Humanoid at 10,000	552 K	-	-	3,260 K	-	-	-	5.91	-
Ant at 1,000	21 K	28 K	62 K	185 K	42 K	1.33	2.95	8.81	2.00
Ant at 3,000	56 K	56 K	152 K	940 K	79K	1.00	2.71	16.79	1.41
Ant at 6,000	248 K	-	-	3,055 K	-	-	-	12.31	-
Walker at 1,000	27 K	42 K	54 K	110 K	50 K	1.56	2.00	4.07	1.85
Walker at 3,000	53 K	79 K	86 K	270 K	89K	1.49	1.62	10.75	1.68
Walker at 5,000	147 K	272 K	-	960 K	270 K	1.85	-	6.53	1.84

Sample efficiency (Chen et al., 2021; Dorner, 2021) is measured by the ratio of the number of samples collected when RAC and some algorithms reach the specified performance. The last four rows show how many times RAC is more sample efficient than other algorithms in achieving that performance.

#### 6.2.3. Value approximation analysis

**Figure 6:**
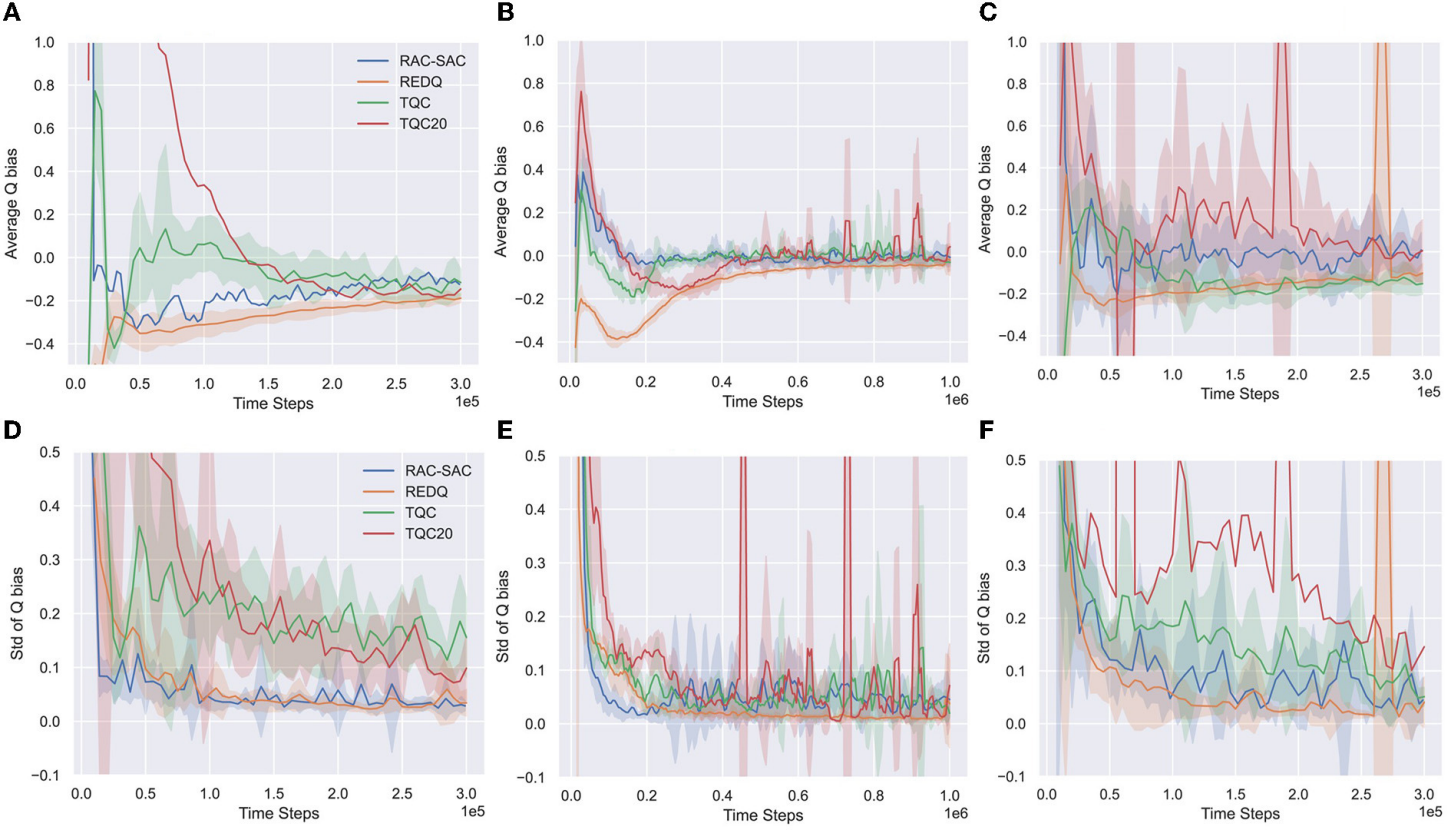
Estimated mean and standard deviation of normalized Q bias of RAC-SAC, REDQ, TQC, and TQC20 for Ant and Humanoid with Monte Carlo method. **(A)** Q bias of Ant, **(B)** Q bias of Humanold, **(C)** Q bias of Walker2d, **(D)** Q standard deviation of Ant, **(E)** Q standard deviation of Humanold, and **(F)** Q standard deviation of Walker2d.

Figure 6 presents the results for Ant, Humanoid, and Walker2d.

In Ant and Walker2d, TQC20 has a high normalized mean of bias, indicating that TQC20 prevents catastrophic overestimation failure accumulation. TQC20 also has a high normalized standard deviation of bias, indicating that the bias is highly non-uniform, which can be detrimental. Since distributional RL is prone to overfitting with few samples, it may not be appropriate to use a high UTD ratio for TQC. In Humanoid, which has a high-dimensional state, overfitting still exists but has been alleviated.

Relative to TQC and TQC20, REDQ and RAC-SAC have a very low normalized standard deviation of bias for most of the training, indicating the bias across different state-action pairs is about the same. Thus, the Q-estimation of REDQ is too conservative in Humanoid, and the large negative bias makes REDQ trapped in a bad locally optimal policy, suffering from pessimistic underexploration. For Ant and Walker2d, although this poor exploration does not harm the performance of the policy, it still slows down convergence speed compared to RAC.

Relative to REDQ, RAC-SAC keeps the Q bias nearly zero without overestimation accumulation; this benign overestimation bias significantly improves performance. RAC-SAC strikes a good balance between overestimation bias (good performance without being trapped in a bad local optimum) and underestimation bias (slight overestimation bias and consistently small standard deviation of bias).

### 6.3. Why Humanoid is hard for most baselines?

**Figure 7:**
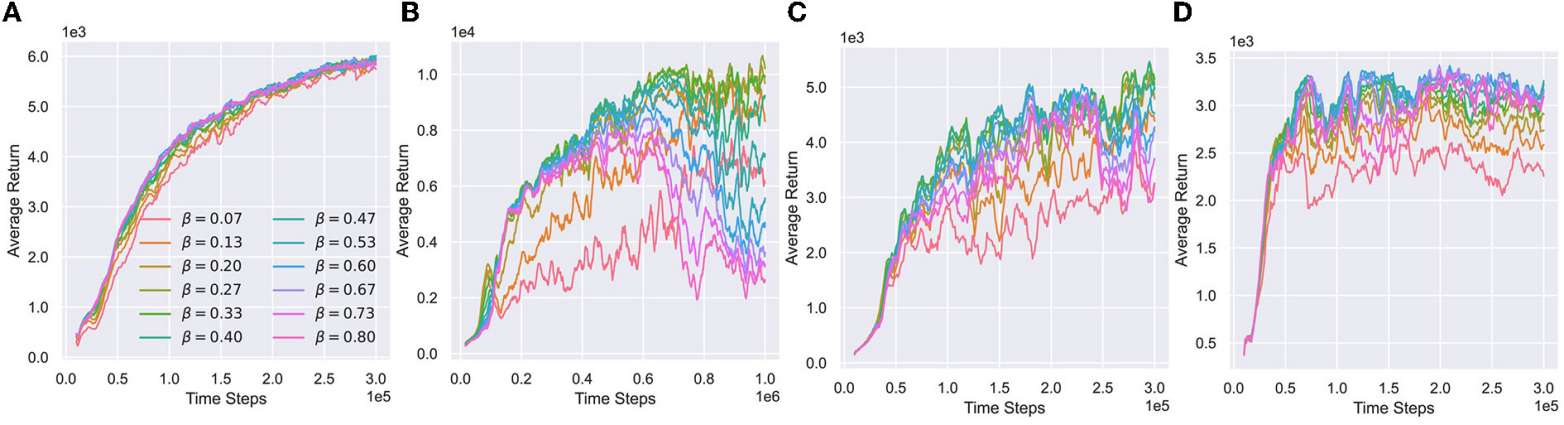
Performance of various value confidence bounds with respect to different β. **(A–D)** Performance respect to different β in Ant, Humanoid, Walker2d, and Hopper. We visualize different β belonging to training distribution $U_{1}=U\left[0,a\right]$ during training processes.

Figure 7 visualizes the performance with respect to various value confidence bounds. Humanoid is extremely sensitive to the value bias. The huge state-action space of Humanoid leads to a large approximation error of the value function with small samples. The approximate lower bound inevitably has spurious maxima, while a small overestimated bias can seriously destabilize updates. It is hard to choose appropriate confidence bound for Humanoid by tuning the hyperparameters, resulting in a difficult under-/overestimation trade-off.

Algorithms (like REDQ) that rely on constant hyperparameters to control the value bias have to conservatively introduce a large underestimation error (Figure 6) to stabilize updates, leading the policy to fall into pessimistic underexploration. In contrast, other algorithms (such as TQC20) plagued by overestimation and overfitting require more samples.

Compared to Humanoid, the state-action space of other environments is much smaller. The approximate Q-functions can easily fit the true Q values accurately, significantly reducing the possibility of spurious maxima. Therefore, optimistic exploration may not be a required component for these environments. So, we can see that they are not very sensitive to various value confidence bounds from Figure 7. An underestimated value is enough to guide the policy to learn stably.

### 6.4. Variants of RAC

**Figure 8:**
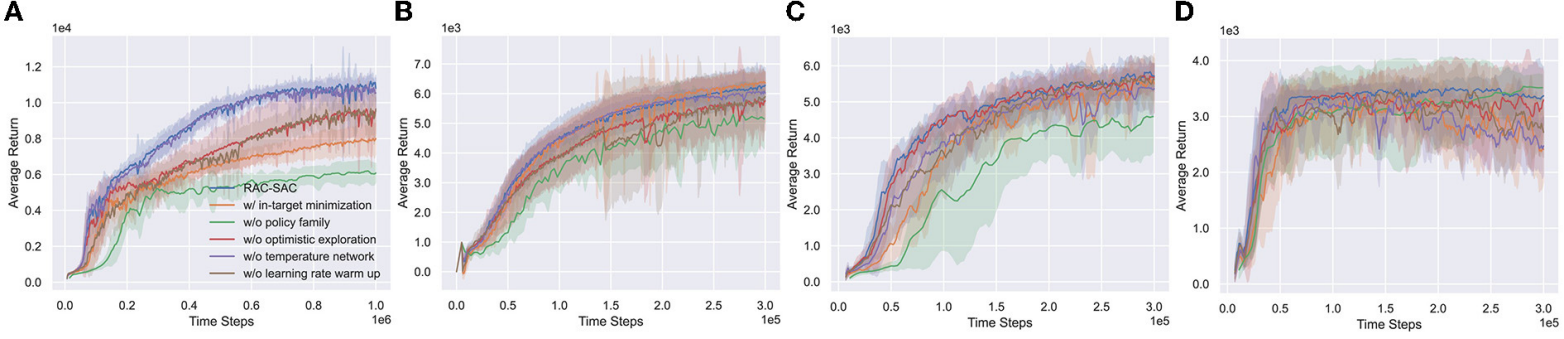
Performance of RAC and its variants. **(A–D)** Humanoid, Ant, Walker2d, and Hopper. The in-target minimization version of RAC is shown in Appendix B.4. RAC without policy family is named Vanilla RAC (see Appendix B.3 for more details. In this case, OAC (Ciosek et al., 2019) is used as optimistic exploration method).

We evaluate the performance contributions of ingredients of RAC (punished Bellman backup, policy family, optimistic exploration, independent temperature network, and learning rate warm-up) on a subset of four environments (see Figure 8).

#### 6.4.1. Punished Bellman backup

When using the in-target minimization instead of punished Bellman backup, RAC is stable, but the performance is significantly worse in Humanoid. Punished Bellman backup provides more finer-grained bias control than in-target minimization, reducing the difficulty of learning representations. Compared with other environments, Humanoid has stronger requirements for state representation learning (Chen et al., 2021). Thus, punished Bellman backup far outperforms in-target minimization in Humanoid and is almost the same in other environments.

#### 6.4.2. Policy family

The policy family is paramount to performance. This is consistent with Section 5.3's conjecture. Even with OAC, an agent can only converge to a local optimum without the policy family in Humanoid, indicating that a single optimistic exploration method cannot solve the pessimistic underexploration well. In addition, the convergence speed of the policy has decreased in Walker2d and Ant.

#### 6.4.3. Optimistic exploration

Experimental results support the point in Section 6.3. Optimistic exploration can help the agent escape from local optima in Humanoid. However, in simple environments (like Ant, Walker2d, and Hopper), optimistic exploration has little impact on performance.

#### 6.4.4. Independent temperature network

Except for Walker2d, the independent temperature network has little effect on RAC performance. The learned temperatures are shown in Appendix C. In practice, we find that the independent temperature network can control the entropy of the policy more quickly and stably.

#### 6.4.5. Learning rate warm-up

A high UTD ratio can easily lead to an excessive accumulation of overestimation errors in the early stage of learning. The learning rate warm-up can alleviate this problem and stabilize the learning process. Without the learning rate warm-up, RAC learns slower at the beginning of the training process.

### 6.5. Hyperparameter ablations

**Figure 9:**
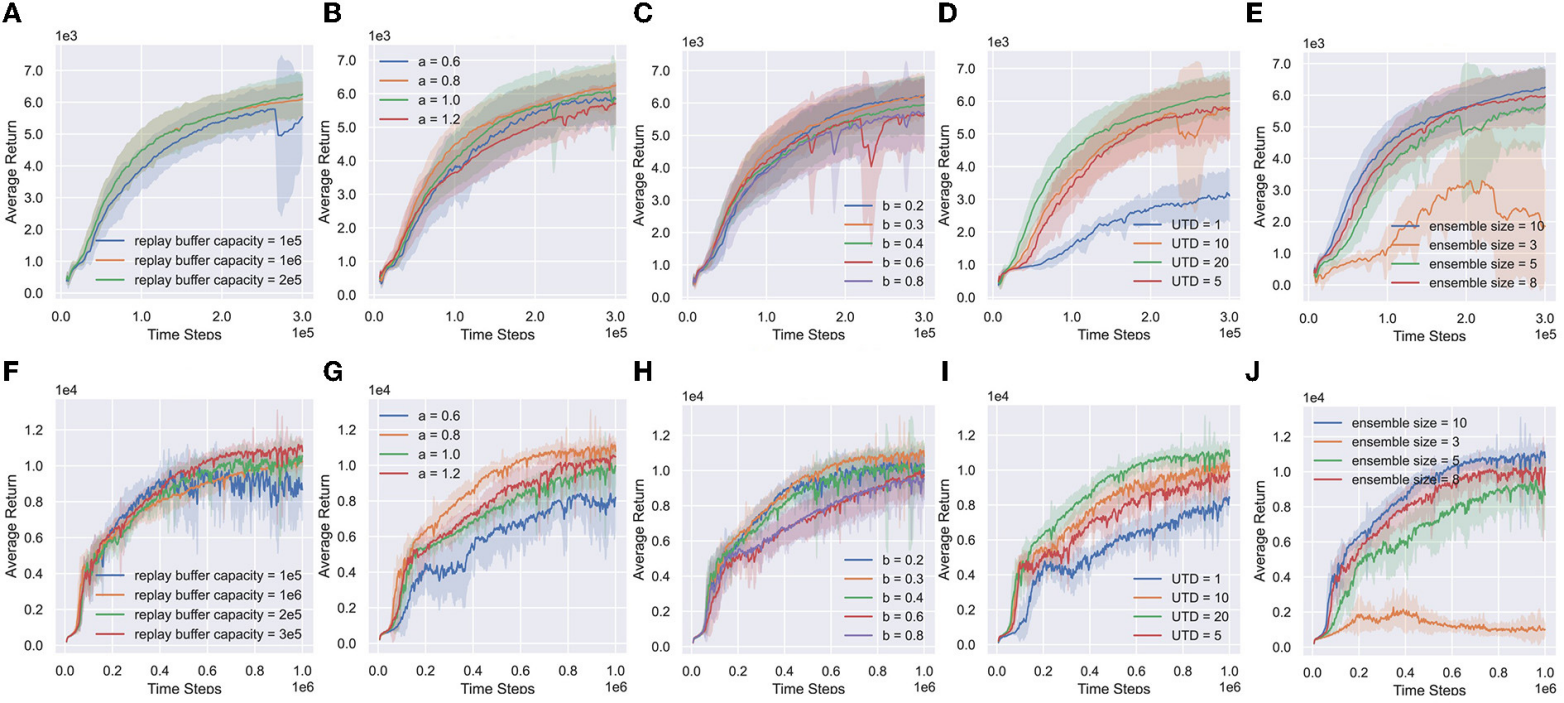
Hyperparameter ablations of RAC. **(A–E)** Replay buffer capacity, right side of exploitation distribution (a), right side of exploration distribution (b), the UTD ratio and the ensemble size for ant. **(F–J)** Replay buffer capacity, right side of exploitation distribution (a), right side of exploration distribution (b), the UTD ratio and the Ensemble size for Humanoid.

RAC introduces some hyperparameters: (1) replay buffer capacity; (2) right side of exploitation distribution *U*_1_ (*a*); (3) right side of exploration distribution *U*_2_ (*b*); (4) UTD ratio G in Algorithm 1; and (5) Ensemble size. Figure 9 shows the numerical results.

Replay buffer capacity (Figures 9A, F). RAC can benefit from a smaller capacity but will be hurt when the capacity is excessively small.

The right side of *U*_1_ (*a*) (Figures 9B, G). *a* is a key hyperparameter of RAC. Because *a* controls the underestimation bias of RAC, which determines the lower bound of Q-functions. The learning process becomes stable with *a* increasing. However, if *a* is too large, it will reduce the learning opportunity of optimistic policies, thereby reducing the learning efficiency.

The right side of *U*_2_ (*b*) (Figures 9C, H). Exploration policies become more conservative with *b* increasing, and the performance of RAC gradually declines. The increasing standard deviation means that more and more agents fall into local-optimal policies. However, if *b* is too small, policies may over-explore the overestimated state, resulting in a decrease in learning efficiency.

The ensemble size (Figures 9E, J) and the UTD ratio (Figures 9D, I). RAC appears to benefit greatly from the ensemble size and UTD ratio. When the ensemble size and UTD ratio are increased, we generally get a more stable average bias, a lower standard deviation of bias, and stronger performance.

## 7. Conclusion

In this study, we empirically discussed under-/ overestimation trade-off on improving the sample efficiency in DRL and proposed the Realistic Actor-Critic (RAC), which learns together values and policies with different trade-offs between underestimation and overestimation in the same network. This study proposed Punished Bellman backup that provides fine-granular estimation bias control to make value approximation smoothly shift between upper bounds and lower bounds. This study also discussed the role of the various components of RAC. Experiments show advantageous properties of RAC: low-value approximation error and brilliant sample efficiency. Furthermore, continuous control benchmarks suggest that RAC consistently improves performances and sample efficiency of existing off-policy RL algorithms, such as SAC and TD3. It is of great significance for promoting reinforcement learning in the robot control domain.

Our results suggest that directly incorporating uncertainty to value functions and learning a powerful policy family can provide a promising avenue for improved sample efficiency and performance. Further exploration of ensemble methods, including high-level policies or more rich policy classes, is an exciting avenue for future work.

## Data availability statement

The original contributions presented in the study are included in the article/Supplementary material, further inquiries can be directed to the corresponding author.

## Author contributions

SL implemented the code and drafted the manuscript. QT assisted in implementing the code and discussed the manuscript. YP assisted in implementing the code and discussed the manuscript. XM guided the research and discussed the results. GW guided the research, implemented parts of the code, and revised the manuscript. All authors contributed to the article and approved the submitted version.
