# Supplementary material for: Realistic Actor-Critic: A framework for balance between value overestimation and underestimation
Source: Front Neurorobot. 2023 Jan 9;16:1081242. doi: 10.3389/fnbot.2022.1081242 (PMC9868235; doi:10.3389/fnbot.2022.1081242)
Supplement: Supplementary file 1 [file Presentation_1.pdf]

APPENDIX

A EXPERIMENTS SETUPS AND METHODOLOGY

A.1 Evaluation method

For all training instances, the policies are evaluated every $R_{eval} = 10^3$ time steps. The agent fixes its policy at each evaluation phase and deterministically interacts with the same environment, separate from obtaining 10 episodic rewards. The mean and standard deviation of these 10 episodic rewards is the performance metrics of the agent at the evaluation phase.

In the case of RAC, we employ a discrete number H of values $\{\beta_i\}_{i=1}^{H}$ to get H policies:

$$\beta_i = b/H \cdot i, i = 1, \dots, H. \tag{S1}$$

Each H policy fixes its policy at the evaluation phase and deterministically interacts with the environment with the fixed policy to obtain 10 episodic rewards. First, the 10 episodic rewards are averaged for each policy, and then the maximum of the 10-episode-average rewards of the H policies is taken as the performance at that evaluation phase.

We performed this operation for 8 different random seeds used in the computational packages(NumPy (Van Der Walt et al., 2011), PyTorch (Paszke et al., 2019)) and environments(OpenAI gym (Brockman et al., 2016)). The mean and standard deviation of the learning curve are obtained from these 8 simulations.

A.2 The normalized value bias estimation

Given a state-action pair, the normalized value bias is defined as:

$$\left(\bar{Q}_\theta(\mathbf{s}, \mathbf{a}) - Q^\pi(\mathbf{s}, \mathbf{a})\right) / \left|E_{\bar{\mathbf{s}},\bar{\mathbf{a}}\sim\pi}\left[Q^\pi(\bar{\mathbf{s}}, \bar{\mathbf{a}})\right]\right|, \tag{S2}$$

where

- $Q^\pi(\mathbf{s}, \mathbf{a})$ be the action-value function for policy π using the standard infinite-horizon discounted Monte Carlo return definition.
- $\bar{Q}_\theta(\mathbf{s}, \mathbf{a})$ the estimated Q-value, defined as the mean of $Q_{\theta_i}(\mathbf{s}, \mathbf{a}), i = 1, \dots, N$.

For RAC, the normalized value bias is defined as:

$$\left(\bar{Q}_\theta(\mathbf{s}, \mathbf{a}, \beta^*) - Q^{\pi^*}(\mathbf{s}, \mathbf{a})\right) / \left|E_{\bar{s},\bar{a}\sim\pi^*}\left[Q^{\pi^*}(\bar{\mathbf{s}}, \bar{\mathbf{a}})\right]\right|, \tag{S3}$$

where

- π^* is the best-performing policy in the evaluation among H policies A.1.
- $Q^{\pi^*}(\mathbf{s}, \mathbf{a})$ be the action-value function for policy π^* using the standard infinite-horizon discounted Monte Carlo return definition.
- $\bar{Q}_\theta(\mathbf{s}, \mathbf{a}, \beta^*)$ the estimated Q-value using Q_θ of β^* which corresponds to the policy π^*, defined as the mean of $Q_{\theta_i}(\mathbf{s}, \mathbf{a}, \beta^*), i = 1, \dots, N$.

To get various target state-action pairs, we first execute the policy in the environment to obtain 100 state-action pairs and then sample the target state-action pair without repetition. Starting from the target state-action pair, run the Monte Carlo processes until the max step limit is reached.

B HYPERPARAMETERS AND IMPLEMENTATION DETAILS

We implement all RAC algorithms with Pytorch (Paszke et al., 2019) and use Ray[tune] (Liaw et al., 2018) to build and run distributed applications. For all the algorithms and variants, we first obtain 5000 data points by randomly sampling actions from the action space without making any parameter updates. Then, to stabilize the early learning of critics, a linear learning rate warm-up strategy is applied to critics in the start stage of training for RAC and its variants:

$$l = l_{init}(1-p) + p \cdot l_{target}, p = \text{clip}(\frac{t - t_{start}}{t_{target} - t_{start}}, 0, 1), \tag{S4}$$

where l is current learning rate, l_{init} is the initial value of the learning rate, l_{target} is the target value of the learning rate, t_{start} is the time steps to start adjusting the learning rate, t_{target} is the time steps to arrive at l_{target}.

For all RAC algorithms and variants, We parameterize both the actor and critics with feed-forward neural networks with 256 and 256 hidden nodes, respectively, with rectified linear units (ReLU) (Nair and Hinton, 2010) between each layer. β is log-scaled before input into actors and critics. In order to prevent β sample from being zero, a small value $\varepsilon = 10^{-7}$ is added to the left side of U_1 and U_2 to be $U_1[\varepsilon, a]$ and $U_2[\varepsilon, b]$. Weights of all networks are initialized with Kaiming Uniform Initialization (He et al., 2015), and biases are zero-initialized. We normalize actions to a range of $[-1, 1]$ for all environments.

B.1 RAC-SAC algorithm

Here, the policy is modeled as a Gaussian with mean and covariance given by neural networks to handle continuous action spaces. The way RAC optimizes the policy makes use of the reparameterization trick (Kingma and Welling, 2013; Haarnoja et al., 2018), in which a sample is drawn by computing a deterministic function of the state, policy parameters, and independent noise:

$$\tilde{\mathbf{a}}_\phi(\mathbf{s}, \beta, \xi) = \tanh\left(\mu_\phi(\mathbf{s}, \beta) + \sigma_\phi(\mathbf{s}, \beta) \odot \xi\right), \quad \xi \sim \mathcal{N}(0, I). \tag{S5}$$

The actor network outputs the Gaussian's means and log-scaled covariance, and the log-scaled covariance is clipped in a range of $[-10, 2]$ to avoid extreme values. Then, the actions are bounded to a finite interval by applying an invertible squashing function ($tanh$) to the Gaussian samples, and the Squashed Gaussian Trick (Haarnoja et al., 2018) calculates the log-likelihood of actions.

The temperature is parameterized by a one-layer feedforward neural network T_ψ of 64 with rectified linear units (ReLU). To prevent temperature be negative, we parameterize temperature as:

$$\alpha_\psi(\beta) = e^{T_\psi(\log(\beta)) + \xi}, \tag{S6}$$

where ξ is constant controlling the initial temperature, $\log(\beta)$ is log-scaled β, $T_\psi(\log(\beta))$ is the output of the neural network.

Algorithm 1 RAC: TD3 version

Initialize actor network ϕ, N critic networks $\theta_i, i = 1, \ldots, N$, empty replay buffer $\mathcal{B}$, target network $\bar{\theta}_i \longleftarrow \theta_i$, for $i = 1, 2, \ldots, N$, uniform distribution $\mathcal{U}_1$ and U_2
for each iteration **do**
  execute an action:
  $\mathbf{a} = \pi_\phi(\cdot \mid \mathrm{s}, \beta) + \epsilon, \epsilon \sim \mathcal{N}(0, \sigma), \beta \sim U_2$.
  Observe reward r, new state s'
  Store transition tuple $\mathcal{B} \leftarrow \mathcal{B} \cup \{(\mathrm{s}, \mathbf{a}, r, \mathrm{s}')\}$
  for G updates **do**
    Sample random minibatch:
    $\{\tau_j\}_{j=1}^{B} \sim \mathcal{B}$, $\{\beta_m\}_{m=1}^{B} \sim U_1$
    Compute the Q target (S7)
    for $i = 1, ..., N$ **do**
      Update θ_i by minimize $\mathcal{L}_{\texttt{critic}}^{\texttt{RAC}}$
      Update target networks:
      $\bar{\theta}_i \leftarrow \rho\bar{\theta}_i + (1 - \rho)\theta_i$
  Update ϕ by minimize $\mathcal{L}_{\texttt{actor}}^{\texttt{RAC-TD3}}$ (S10)

B.2 RAC-TD3 algorithm

We implement RAC-TD3 referring to `https://github.com/sfujim/TD3`. A final *tanh* unit following the output of the actor. Unlike TD3, we did not use a target network for the actor and delayed policy updates. For each update of critics, a small amount of random noise is added to the policy and averaged over mini-batches:

$$y = r + \gamma \left[\bar{Q}_{\bar{\theta}}(\mathrm{s}', \mathbf{a}', \beta) - \beta \hat{s}(Q_{\bar{\theta}}(\mathrm{s}', \mathbf{a}', \beta))\right], \tag{S7}$$

$$\mathbf{a}' = \mathrm{clip}(\pi_\phi\left(\cdot \mid \mathrm{s}', \beta\right) + \epsilon, -1, 1), \tag{S8}$$

$$\epsilon \sim \mathrm{clip}(\mathcal{N}(0, \sigma), -c, c). \tag{S9}$$

The extended policy π_ϕ is updated by minimizing the following object:

$$\mathcal{L}_{\texttt{actor}}^{\texttt{RAC-TD3}}(\phi) = \mathbb{E}_{\mathrm{s}\sim\mathcal{B}, \beta\sim U_1}\left[-\bar{Q}_\theta\left(\mathbf{a}, \mathrm{s}, \beta\right)\right], \mathbf{a} = \pi_\phi\left(\cdot \mid \mathrm{s}, \beta\right). \tag{S10}$$

The pseudocode for RAC-TD3 is shown in Algorithm 1.

B.3 Vanilla RAC algorithm

UVFA is not needed for vanilla RAC as β is a constant. The actor is updated by minimizing the following object:

$$\mathcal{L}_{\texttt{actor}}^{\texttt{vanillaRAC}}(\phi) = \mathbb{E}_{\mathrm{s}\sim\mathcal{B}, \mathbf{a}\sim\pi_\phi}\left[\alpha \log\left(\pi_\phi\left(\mathbf{a} \mid \mathrm{s}\right)\right) - \bar{Q}_\theta\left(\mathbf{a}, \mathrm{s}\right)\right]. \tag{S11}$$

The pseudocode for Vanilla RAC is shown in Algorithm 2.

B.4 RAC with in-target minimization

We implement RAC with in-target minimization referring to authors's code `https://github.com/watchernyu/REDQ`. The critics and actor are extended as $Q_{\theta_i}(\mathrm{s}, \mathbf{a}, k)$ and $\pi_\phi\left(\cdot \mid \mathrm{s}', k\right)$, U_1 is a uniform traning distribution $\mathcal{U}[1, a]$, $a > 1$, $k \sim U_1$ that determine the size of the random subset $\mathcal{M}$. When k is

Algorithm 2 Vanilla RAC

Initialize actor network ϕ, N critic networks $\theta_i, i=1,\ldots,N$, empty replay buffer $\mathcal{B}$, target network $\bar{\theta_i} \longleftarrow \theta_i$, for $i=1,2,\ldots,N$
for each iteration **do**
  execute an action $\mathbf{a} \sim \pi_\phi(\cdot \mid s)$.
  Observe reward r, new state s'
  Store transition tuple $\mathcal{B} \leftarrow \mathcal{B} \cup \{(\mathrm{s}, \mathbf{a}, r, \mathrm{s}')\}$
  for G updates **do**
    Sample random minibatch $\{\tau_j\}_{j=1}^{B} \sim \mathcal{B}$
    Compute the Q target
    for $i=1,\ldots,N$ **do**
      Update θ_i by minimize $\mathcal{L}_{\texttt{critic}}$
      Update target networks:
      $\bar{\theta_i} \leftarrow \rho\bar{\theta_i} + (1-\rho)\theta_i$
  Update ϕ by minimize $\mathcal{L}_{\texttt{actor}}^{\texttt{vanillaRAC}}$ (S11)
  Update α by minimize $\mathcal{L}_{\texttt{temp}}$

not an integer, the size of $\mathcal{M}$ will be sample beweetn $\mathrm{floor}(k)$ and $\mathrm{floor}(k+1)$ according to the Bernoulli distribution $\mathcal{B}(p)$ with parameter $p = k - \mathrm{floor}(k)$, where floor is a round-towards-zero operator.

An independent temperature network α_ψ parameterized by ψ is updated with the following object:

$$\mathcal{L}_{\texttt{temp}}^{\texttt{RAC}}(\psi) = \mathbb{E}_{\mathrm{s}\sim\mathcal{B},\mathbf{a}\sim\pi_\phi,k\sim U_1}[-\alpha_\psi(k)\log\pi_\phi(\mathbf{a} \mid \mathrm{s}, k) - \alpha_\psi(k)\overline{\mathcal{H}}]. \tag{S12}$$

In-target minimization is used to calculate the target y:

$$y = r + \gamma\mathbb{E}_{\mathbf{a}'\sim\pi_\phi}[\min_{i\in\mathcal{M}} Q_{\bar{\theta}_i}\left(\mathrm{s}', \mathbf{a}', k\right) - \alpha_\psi(k)\log\pi_\phi\left(\mathbf{a}' \mid \mathrm{s}', k\right)], \tag{S13}$$

Then each $Q_{\theta_i}(\mathrm{s}, \mathbf{a}, k)$ is updated with the same target:

$$\mathcal{L}_{\texttt{critic}}^{\texttt{RAC}}(\theta_i) = \mathbb{E}_{\tau_t\sim\mathcal{B},k\sim U_1}[(Q_{\theta_i}(\mathrm{s}, \mathbf{a}, k) - y)^2]. \tag{S14}$$

The extended policy π_ϕ is updated by minimizing the following object:

$$\mathcal{L}_{\texttt{actor}}^{\texttt{RAC}}(\phi) = \mathbb{E}_{\mathrm{s}\sim\mathcal{B},k\sim U_1}[\mathbb{E}_{\mathbf{a}\sim\pi_\phi}[\alpha_\psi(k)\log\left(\pi_\phi(\mathbf{a} \mid \mathrm{s}, k)\right) - \bar{Q}_\theta(\mathbf{a}, \mathrm{s}, k)]]. \tag{S15}$$

When interacting with the environment, obtaining exploration behaviors by sample k from exploration distribution $U_2 = \mathcal{U}[1, b]$, $a > b > 1$.

The pseudocode for RAC with in-target minimization is shown in Algorithm 3.

B.5 Hyperparameter setting

Table S3 and S2 lists the hyperparameters for RAC and variants used in experiments.

Algorithm 3 RAC with in-target minimization

Initialize actor network ϕ, N critic networks $\theta_i, i = 1, \ldots, N$, temperature network ψ, empty replay buffer $\mathcal{B}$, target network $\bar{\theta}_i \longleftarrow \theta_i$, for $i = 1, 2, \ldots, N$, uniform distribution U_1 and U_2
for each iteration **do**
    execute an action $\mathbf{a} \sim \pi_\phi(\cdot \mid \mathrm{s}, k), k \sim U_2$.
    Observe reward r, new state s'
    Store transition tuple $\mathcal{B} \leftarrow \mathcal{B} \cup \{(\mathrm{s}, \mathbf{a}, r, \mathrm{s}')\}$
    for G updates **do**
        Sample random minibatch:
        // UPDATE CRITICS VIA IN-TARGET MINIMIZATION
        $\{\tau_j\}_{j=1}^{B} \sim \mathcal{B}$, $\{k_j\}_{j=1} \sim U_1$
        Sample a set $\mathcal{M}$ of k distinct indices from $\{1, 2, \ldots, N\}$
        Compute the Q target (S13)
        for $i = 1, \ldots, N$ **do**
            Update θ_i by minimize $\mathcal{L}_{\texttt{critic}}^{\texttt{RAC}}$ (S14)
            Update target networks:
            $\bar{\theta}_i \leftarrow \rho\bar{\theta}_i + (1-\rho)\theta_i$
    Update ϕ by minimize $\mathcal{L}_{\texttt{actor}}^{\texttt{RAC-SAC}}$ (S15)
    Update ψ by minimize $\mathcal{L}_{\texttt{temp}}^{\texttt{RAC}}$ (S12)

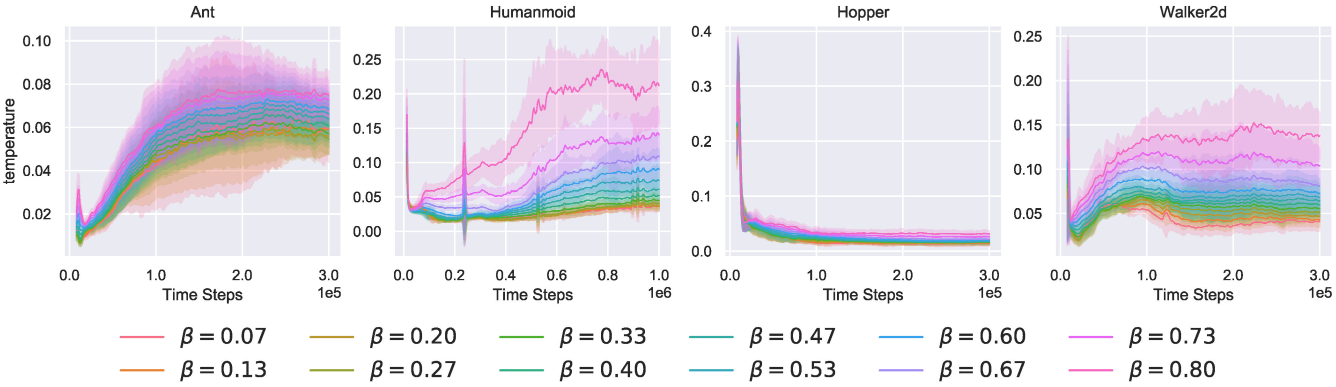

Figure S1: Visualisations of learned temperatures for RAC-SAC with different β.

C VISUALISATIONS

learned temperatures. The Figure S1 shows the visualization of learned temperatures concerning different β during training. The figure demonstrates that learned temperatures are quite different. It is challenging to consider the temperature of different β with a single temperature.

REFERENCES

Brockman, G., Cheung, V., Pettersson, L., Schneider, J., Schulman, J., Tang, J., et al. (2016). Openai gym. *arXiv preprint arXiv:1606.01540*

Haarnoja, T., Zhou, A., Hartikainen, K., Tucker, G., Ha, S., Tan, J., et al. (2018). Soft actor-critic algorithms and applications. *arXiv preprint arXiv:1812.05905*

He, K., Zhang, X., Ren, S., and Sun, J. (2015). Delving deep into rectifiers: Surpassing human-level performance on imagenet classification. In *Proceedings of the IEEE international conference on computer vision*. 1026–1034

Table S1. Environment dependent hyperparameters.

Hyperparameters	Humanoid	Walker	Ant	Hopper	HalfCheetah	Swimmer
RAC-SAC						
replay buffer capacity	3×10^5	10^5	2×10^5	1×10^6	2.5×10^5	2×10^5
exploitation distribution U_1	$\mathcal{U}[10^{-7}, 0.8]$	$\mathcal{U}[10^{-7}, 0.8]$	$\mathcal{U}[10^{-7}, 0.8]$	$\mathcal{U}[10^{-7}, 0.8]$	$\mathcal{U}[10^{-7}, 0.3]$	$\mathcal{U}[10^{-7}, 0.8]$
exploration distribution U_1	$\mathcal{U}[10^{-7}, 0.3]$	$\mathcal{U}[10^{-7}, 0.3]$	$\mathcal{U}[10^{-7}, 0.3]$	$\mathcal{U}[10^{-7}, 0.3]$	$\mathcal{U}[10^{-7}, 0.1]$	$\mathcal{U}[10^{-7}, 0.3]$
RAC-TD3						
replay buffer capacity	3×10^5	10^5	2×10^5	1×10^6	2.5×10^5	2×10^5
exploitation distribution U_1	$\mathcal{U}[10^{-7}, 0.8]$	$\mathcal{U}[10^{-7}, 0.8]$	$\mathcal{U}[10^{-7}, 0.8]$	$\mathcal{U}[10^{-7}, 0.8]$	$\mathcal{U}[10^{-7}, 0.3]$	$\mathcal{U}[10^{-7}, 0.8]$
exploration distribution U_1	$\mathcal{U}[10^{-7}, 0.3]$	$\mathcal{U}[10^{-7}, 0.3]$	$\mathcal{U}[10^{-7}, 0.3]$	$\mathcal{U}[10^{-7}, 0.3]$	$\mathcal{U}[10^{-7}, 0.1]$	$\mathcal{U}[10^{-7}, 0.3]$
Vanilla RAC						
replay buffer capacity	10^6	10^6	10^6	10^6	10^6	10^6
uncertainty punishment (β)	0.2	0.3	0.2	0.2	0.1	0.4
RAC-in-target						
replay buffer capacity	3×10^5	10^5	2×10^5	1×10^6	1×10^6	1×10^6

Table S2. Environment dependent hyperparameters.

Hyperparameters	Value
RAC-SAC	
initial temperature coefficient (ξ)	-5
RAC-TD3	
exploration noisy	$\mathcal{N}(0, 0.1)$
policy noisy (σ)	0.2
policy noisy clip (c)	0.5
Vanilla RAC	
initial temperature	$\exp(-3)$
uncertainty punishment (β)	0.3
RAC with in-target minimization	
initial temperature coefficient (ξ)	-5
exploitation distribution U_1	$\mathcal{U}[1, 1.5]$
exploration distribution U_2	$\mathcal{U}[1, 2.0]$

Kingma, D. P. and Ba, J. (2014). Adam: A method for stochastic optimization. *arXiv preprint arXiv:1412.6980*

Kingma, D. P. and Welling, M. (2013). Auto-encoding variational bayes. *arXiv preprint arXiv:1312.6114*

Liaw, R., Liang, E., Nishihara, R., Moritz, P., Gonzalez, J. E., and Stoica, I. (2018). Tune: A research platform for distributed model selection and training. *arXiv preprint arXiv:1807.05118*

Nair, V. and Hinton, G. E. (2010). Rectified linear units improve restricted boltzmann machines. In *Icml*

Paszke, A., Gross, S., Massa, F., Lerer, A., Bradbury, J., Chanan, G., et al. (2019). Pytorch: An imperative style, high-performance deep learning library. *Advances in neural information processing systems* 32

Van Der Walt, S., Colbert, S. C., and Varoquaux, G. (2011). The numpy array: a structure for efficient numerical computation. *Computing in science & engineering* 13, 22–30

Table S3. Shared hyperparameters

Hyperparameters	Value
optimizer	Adam (Kingma and Ba, 2014)
actor learning rate	3×10^{-4}
temperature learning rate	3×10^{-4}
initial critic learning rate (l_{init})	3×10^{-5}
target critic learning rate (l_{target})	3×10^{-4}
time steps to start learning rate adjusting (t_{start})	5000
time steps to reach target learning rate (t_{target})	10^4
number of hidden layers (for ϕ and θ_i)	2
number of hidden units per layer (for ϕ and θ_i)	256
number of hidden layers (for T_ψ)	1
number of hidden units per layer (for T_ψ)	64
discount (γ)	0.99
nonlinearity	ReLU
evaluation frequence	10^3
minibatch size	256
target smoothing coefficient (ρ)	0.005
Update-To-Data (UTD) ratio (G)	20
ensemble size (N)	10
number of evaluation episodes	10
initial random time steps	5000
frequence of delayed policy updates	1
log-scaled covariance clip range	$[-10, 2]$
number of discrete policies for evaluation (H)	12
